# The role of lifestyle, health, and work in educational inequalities in sick leave and productivity loss at work

**DOI:** 10.1007/s00420-012-0793-1

**Published:** 2012-07-07

**Authors:** Suzan J. W. Robroek, Frank J. van Lenthe, Alex Burdorf

**Affiliations:** Department of Public Health, Erasmus MC, University Medical Center Rotterdam, PO Box 2040, 3000 CA Rotterdam, The Netherlands

**Keywords:** Productivity loss at work, Sick leave, Education, Inequalities

## Abstract

**Purpose:**

To investigate the influence of lifestyle, health, and work conditions in the association between education and productivity loss at work and sick leave.

**Methods:**

Employees of six companies filled out a questionnaire on demographics, lifestyle-related, health, and work-related factors, and productivity loss at work and sick leave at baseline (*n* = 915) and after 1-year (*n* = 647).

**Results:**

Employees with a low education were more likely to report productivity loss at work (OR = 1.49, 95 % CI 0.98–2.26) and sick leave (OR = 1.81, 95 % CI 1.15–2.85). After adjustment for lifestyle, health, and work conditions, the association between education and productivity loss at work did not attenuate. Work conditions attenuated the association between low education and sick leave (OR = 1.62, 95 % CI 1.01–2.61), and additional adjustment for health and lifestyle-related factors further reduced the strength of the association (OR = 1.42, 95 % CI 0.86–2.34).

**Conclusion:**

Work conditions and lifestyle-related factors partly explained the association between education and sick leave, but did not influence the association between education and productivity loss at work. The educational differences in sick leave prompt for interventions that address behavioral aspects as well as work-related and lifestyle-related factors.

## Introduction

Workers with a low education or working in lower occupational social classes have a higher risk of disability retirement and sick leave (Beemsterboer et al. 2009; Duijts et al. 2007; Leinonen et al. 2011). The mechanisms through which socioeconomic position affects these outcomes are not yet established. Working conditions as well as lifestyle-related factors and health might play a role in the causal pathway of educational inequalities in productivity loss at work and sick leave. Insight into such underlying factors is of importance to design successful interventions to decrease educational inequalities in sick leave.

A low educational level is associated with both strenuous physical and psychosocial working conditions (Schrijvers et al. 1998), which are determinants of both productivity loss at work and sick leave (Alavinia et al. 2009a; Martimo et al. 2009; Moreau et al. 2004). Strenuous working conditions might therefore contribute to educational inequalities in productivity loss at work and sick leave. The role of working conditions on the relation between educational inequalities and sick leave has been studied before. Previous studies found that a substantial part of the relation between lower occupational class and sick leave could be attributed to physical working conditions and a low job control (Laaksonen et al. 2010a; Melchior et al. 2005; Niedhammer et al. 2008). Melchior et al. (2005) reported that a set of working conditions, with both physical and psychosocial work-related factors (e.g., demands, control, social support), accounted for 16 % (men) to 25 % (women) of the occupational class differences in sick leave. Laaksonen et al. (2010a) found that the occupational group differences in sickness absence reduced by about 40 % after adjustment for physical working conditions.

The role of other factors on the relation between educational level and sick leave is less clear. An unhealthy lifestyle and poor health are also more prevalent among individuals with a low education than among better educated individuals (Kamphuis et al. 2008; Kunst et al. 2005; Mackenbach et al. 2008) and have also been found to be associated with productivity loss at work and sick leave (Bernaards et al. 2007; Gates et al. 2008; Laaksonen et al. 2009; Neovius et al. 2009; Pronk et al. 2004; Robroek et al. 2011; Schultz and Edington 2007; Van Duijvenbode et al. 2009). Laaksonen et al. (2009) reported that smoking and overweight explained part of the relation between occupational class and sick leave. However, the role of lifestyle-related factors in potential educational differences in productivity loss at work remains largely unknown.

In summary, little is known on the mechanisms through which socioeconomic factors affect sick leave, and productivity loss at work. In the current study, both lifestyle-related and work-related factors can be analyzed simultaneously to investigate their relative influence on the association between educational level and productivity loss at work and sick leave. It is aimed to get insight into the role of health, lifestyle-related and work-related factors in educational inequalities in productivity loss at work and sick leave.

## Methods

### Study design, participants, and recruitment

Participants were employees from healthcare organizations (*n* = 2), commercial services (*n* = 2), and the executive branch of government (*n* = 2), with the main occupational groups: clerical workers, financial workers, managers, nurses and nursing aides, and policemen. The study took place in The Netherlands between October 2007 and November 2010. Within the participating companies, the study was announced through e-mail, internet, and/or a company magazine. Three companies restricted the maximum number of participants on a ‘first in’ principle. Participants enrolled voluntarily in the study by visiting the study website and completing the baseline questionnaire on lifestyle-related factors, health, work demands, productivity loss at work, and sick leave. Subsequently, they could participate in a physical health check. One year after the baseline measurements, participants were asked to fill out the first follow-up questionnaire. Thirty-six workers were excluded due to working <12 h per week for the company, and an additional 36 did not complete the full questionnaire. Of the 915 participants with baseline information on educational level, lifestyle-related factors, productivity loss at work, and sick leave, 71 % filled out the 1-year follow-up questionnaire (*n* = 647). The Medical Ethics Committee of Erasmus MC, University Medical Center in Rotterdam, The Netherlands, approved the study and all participants gave written informed consent.

### Outcomes

#### Productivity loss at work

At baseline and 1-year follow-up, productivity loss at work was measured with the quantity scale of the Quantity and Quality (QQ) method (Brouwer et al. 1999). This measure showed a moderate correlation with objective work output (*r* = 0.48) among floor layers (Meerding et al. 2005). Respondents were asked to indicate how much work they actually performed during regular hours on their most recent regular workday, compared with normal. The amount of productivity was measured on a scale from 0 (nothing) to 10 (regular amount). The outcome productivity loss at work was classified into three categories: no productivity loss (score = 10), 10–20 % productivity loss (score = 8 or score = 9), and 30 % or more productivity loss at work (score of 7 or lower).

#### Sick leave

Sick leave was derived from the work ability index (WAI) and measured both at baseline and 1-year follow-up (Tuomi et al. 1998). Participants were asked to indicate on a 5-point ordinal scale how many days in the past 12 months they were not able to work due to health problems. The outcome sick leave was classified into three categories: no sick leave, 1–9 days, and 10 days or more with sick leave.

### Determinants

#### Individual characteristics

In the baseline questionnaire, participants were asked about their age, sex, education, and ethnicity. Educational level was assessed by the highest level of education completed and was defined as low (primary school, lower and intermediate secondary schooling, or lower vocational training), intermediate (higher secondary schooling or intermediate vocational schooling), and high (higher vocational schooling or university). Two categories were created for ethnicity: Dutch and other, according to the standardized procedures described by Statistics Netherlands (2004).

#### Lifestyle-related factors

Self-reported lifestyle-related factors were measured both at baseline and at 1-year follow-up. Physical activity (PA) was measured in the baseline questionnaire by the short version of the international physical activity questionnaire (IPAQ), which assessed vigorous and moderate intensity PA (Craig et al. 2003). The average time spent on PA per day was calculated. Walking was not included in this calculation, since casual walking is regarded a light-intensity activity. For all behaviors, a dichotomous variable was calculated for non-compliance with the national recommendations. For insufficient moderate PA, a cut-off point of <30 min of PA per day was used, and for insufficient vigorous PA, a cut-off point of <3 times a week vigorous PA. For insufficient fruit and vegetable intake, the cut-off point was <400 g of fruit and vegetables. Fruit and vegetable intake was measured with the nine-item validated Dutch Food Frequency Questionnaire (Bogers et al. 2004). Smoking was defined as current smoking status, and excessive alcohol use as drinking 15 or more glasses of alcohol per week for women and 22 or more glasses for men.

#### Health indicators

Self-reported health and body mass index (BMI) were measured at baseline and at 1-year follow-up. The first question of the short form-12 (SF-12) questionnaire was used to measure perceived general health and dichotomized into ‘poor or moderate’ and ‘good to excellent’ (Ware et al. 1996). In the physical health check, height and weight were measured to calculate the body mass index (BMI) and to categorize individuals as normal weight (BMI < 25 kg/m^2^), overweight (25 ≤ BMI < 30 kg/m^2^), and obese (BMI ≥ 30 kg/m^2^). In the first follow-up, weight was self-reported in the questionnaire.

#### Work-related factors

The self-reported work-related factors were measured in the baseline questionnaire.

Participants were asked to indicate whether their current job is mainly physically or mentally demanding. In addition, specific psychosocial and physical work demands were asked. The following psychosocial factors were measured with an abbreviated version of a validated Dutch questionnaire about psychosocial job demands on job stress: work demands (6 items, Cronbach’s α = 0.82), job control (4 items, Cronbach’s α = 0.89), skill discretion (4 items, Cronbach’s α = 0.78), and support from colleagues (6 items, Cronbach’s α = 0.74) and supervisor (6 items, Cronbach’s α = 0.79) (Van Veldhoven and Meijman 1994). Questions on work demands were related to excessive work, and insufficient time to complete the work. Job control concerned influence on the planning of tasks, and influence on the pace of work. Skill discretion related to creativity, varied work, and required skills and abilities. Support from colleagues and supervisors was measured with questions related to conflicts, understanding, possibility to ask for help and to count on them, and the atmosphere. For all questions, a four-point scale was used with ratings ‘never’, ‘sometimes’, ‘often’, and ‘always’. A standardized sum score was calculated for each dimension separately, and workers with a score in the upper quartile were regarded as exposed to the psychosocial risk factor.

Physical load in the current job concerned the regular presence of working in awkward postures, and lifting heavy loads. For both factors, a four-point scale was used with rating ‘seldom or never’, ‘now and then’, ‘quite a lot’, and ‘a lot’ during a normal workday. The answers ‘quite a lot’ and ‘a lot’ were classified as high exposure (Elders and Burdorf 2001).

### Statistical analyses

Descriptive statistics were used for characteristics of the study population.

In order to study the association of the dependent variables (‘10–20 % productivity loss at work’ ‘30 % or more productivity loss at work’, ‘1–9 days sick leave’, and’ 10 or more days sick leave’) with educational level, lifestyle-related factors, health, and work-related factors general estimating equations (GEE) were used. GEE is suitable for the analysis of repeated measurements within participants, analyzing the associations between the variables of the model at different time-points simultaneously (Twisk 2003). The absence of productivity loss at work and sick leave were reference categories. In all models, demographic and work-related factors were considered to be time independent, and all associations were adjusted for sex, age, and ethnicity. The associations were adjusted for ethnicity because of its association with educational level, health, and labor force status (Schuring et al. 2009). The odds ratios (OR) were estimated as measure of association with corresponding 95 % confidence intervals (95 % CI).

In order to study the influence of lifestyle-related factors, perceived general health, and work-related factors on the associations between educational levels and productivity loss at work and sick leave, these factors were added separately to the basic statistical model describing the association between educational level and productivity loss at work or sick leave, adjusted for demographic confounders. All variables with an association with educational level (*p* < 0.20) and a statistically significant association with productivity loss at work or sick leave (*p* < 0.05) were selected to study the influence on the association between educational level and productivity loss at work and sick leave. A less stringent significance level was used to identify variables associated with educational level, to avoid that important variables would not end up in the final model. All analyses were carried out with SAS 9.2 statistical software package.

## Results

Table 1 shows that at baseline, 33 % of the subjects reported productivity loss at work during the previous workday and 59 % lost at least one workday because of sick leave in the past 12 months. At 1-year follow-up, 30 % of the participants reported productivity loss at work, and 52 % reported sick leave. Productivity loss at work and sick leave were not associated (Cohen’s κ = 0.07). Productivity loss at work and 10 or more days sick leave were more prevalent among low educated employees as compared to better educated participants. Overweight and obesity and reduced perceived general health were also more prevalent among employees with a low education. Employees with a low educational level more often had physically demanding jobs and jobs with low job control than better educated participants.

**Table 1:** Baseline characteristics of participating employees in 6 companies (*n* = 915)

	Total (*n* = 915)		Low education (*n* = 201)		Intermediate education (*n* = 303)		High education (*n* = 411)	
	*n*	%	*n*	%	*n*	%	*n*	%
*Demographic factors*								
Female gender	469	51	92	46	166	55	211	51
Age (years)*								
<39	376	41	49	24	128	42	199	48
40–49	274	30	66	33	106	35	102	25
50+	265	29	86	43	69	23	110	27
Non-Dutch ethnicity	147	16	49	24	43	14	55	13
*Lifestyle-related factors*								
<30 min/day moderate PA	295	32	80	40	85	28	130	32
<3x/wk 20 min vigorous PA	646	71	144	72	203	67	299	73
<400 g fruit and vegetable intake	429	47	98	49	152	50	179	44
Current smoker	164	18	47	24	49	16	68	17
Excessive alcohol user	24	3	3	2	7	2	14	3
Overweight*	274	34	66	39	95	35	113	31
Obese	70	8	23	14	25	9	22	6
*Health indicator*								
Poor or moderate general health*	58	6	21	10	18	6	19	5
*Work-related factors*								
Physically demanding job*	145	16	51	25	47	16	47	11
Lifting heavy loads	84	9	21	11	28	9	35	9
Awkward postures	117	13	28	14	44	15	45	11
High work demands*	291	32	56	28	89	29	146	36
Low job control*	303	33	75	37	116	38	112	27
Low skill discretion	242	26	49	24	98	32	95	23
Poor relation with colleagues	263	29	47	23	99	33	117	29
Poor relation with supervisor	255	28	49	24	82	27	124	30
*Outcome*								
Productivity loss at work*	302	33	81	40	99	33	122	30
10–20 % productivity loss at work	179	20	49	24	57	19	73	18
≥ 30 % productivity loss at work	123	13	32	16	42	14	49	12
Sick leave	535	59	116	58	192	63	227	55
1–9 days sick leave	404	44	78	39	139	46	187	46
≥ 10 days sick leave*	131	14	38	19	53	17	40	10

*PA * physical activity

* *p* < 0.05 (trend test)

Lifestyle-related, health, and work-related factors were not found to be correlated or were weakly correlated (*r* < 0.30), except the correlations between supervisor and colleague support (*r* = 0.37, *p* < 0.001), between the several physical work demands, and between physical work demands and physical activity (*r* = 0.39, *p* < 0.001).

Twenty-nine percent of the baseline participants were lost to follow-up. Individuals with insufficient fruit and vegetable intake (OR = 0.65, 95 % CI 0.49–0.88) and smokers (OR = 0.53, 95 % CI 0.37–0.75) were less likely to fill out the follow-up questionnaire than workers with a healthy lifestyle. Older employees (OR = 3.01, 95 % CI 1.86–4.86) were more likely to repeat participation at 1-year follow-up.

### Productivity loss at work

As shown in Table 2, participants with a low educational level (OR = 1.49, 95 % CI 0.98–2.26) and participants with insufficient vigorous PA (OR = 1.58, 95 % CI 1.10–2.26) were more likely to report productivity loss at work. The strongest association was found between a poor health and productivity loss at work (OR = 3.24, 95 % CI 1.94–5.41). Low job control (OR = 1.62, 95 % CI 1.16–2.28) and a poor relation with supervisors (OR = 2.16, 95 % CI 1.53–3.05) or colleagues (OR = 1.61, 95 % CI 1.14–2.26) were also associated with productivity loss at work. A statistically significant interaction was found between insufficient vigorous PA and educational level. After stratifying for educational level, insufficient vigorous PA was only associated with 30 % or more productivity loss at work among better educated employees (OR = 3.76, 95 % CI 1.71–8.26). The combination of work-related factors did not explain the association between educational level and productivity loss at work (Table 3). The strength of the association between a low educational level and 30 % or more productivity loss at work was not reduced after adjustment for health, work-related or lifestyle-related factors.

**Table 2:** Univariate odds ratios (OR) and 95 % confidence intervals (95 % CI) of individual characteristics, lifestyle-related and health factors, and work-related factors in relation with productivity loss at work and sick leave among employees in 6 companies (*n* = 647)

		Productivity loss at work				Sick leave			
	Pe	10–20 %^†^ (*n* = 130)		30 % or more^†^ (*n* = 93)		1–9 days^‡^ (*n* = 305)		10 or more days^‡^ (*n* = 97)	
	%	OR	95 % CI	OR	95 % CI	OR	95 % CI	OR	95 % CI
*Educational level*									
Low	21	1.46*	1.01–2.11	1.49	0.98–2.26	1.06	0.76–1.48	1.81*	1.15–2.85
Intermediate	35	1.22	0.89–1.67	1.28	0.87–1.87	1.29	0.98–1.70	1.85*	1.21–2.82
High	45	1.00	–	1.00	–	1.00	–	1.00	–
*Lifestyle-related factors*									
<30 min/day moderate PA	30	1.19	0.90–1.57	1.18	0.83–1.67	0.86	0.68–1.09	0.92	0.65–1.29
<3x/wk 20 min vigorous PA	70	1.08	0.81–1.43	1.58*	1.10–2.26	1.20	0.95–1.52	1.25	0.87–1.81
<400 g fruit and vegetable intake	44	0.85	0.65–1.12	1.00	0.73–1.38	0.95	0.75–1.19	1.12	0.81–1.56
Current smoker	15	1.16	0.81–1.67	0.95	0.62–1.47	1.35	0.97–1.87	1.43	0.93–2.19
Excessive alcohol	3	0.65	0.28–1.53	1.01	0.39–2.66	1.05	0.49–2.22	1.51	0.64–3.60
Overweight	35	1.18	0.87–1.62	1.18	0.83–1.68	1.02	0.79–1.34	1.52*	1.01–2.30
Obese	9	1.12	0.68–1.83	0.79	0.40–1.53	0.76	0.48–1.22	2.29*	1.27–4.12
*Health*									
Poor/moderate general health	6	1.91*	1.10–3.32	3.24*	1.94–5.41	1.87*	1.11–3.16	6.26*	3.47–11.29
*Work-related factors*									
Physically demanding job	15	1.22	0.84–1.77	1.13	0.72–1.77	1.08	0.77–1.53	1.47	0.93–2.32
Lifting heavy loads	9	1.15	0.73–1.81	0.69	0.34–1.38	1.13	0.72–1.76	0.84	0.42–1.68
Awkward postures	13	0.98	0.65–1.81	1.24	0.83–2.26	1.62*	1.09–2.39	2.21*	1.32–3.68
High work demands	31	1.17	0.87–1.57	1.11	0.77–1.60	1.23	0.94–1.61	1.26	0.85–1.87
Low job control	32	1.10	0.82–1.47	1.62*	1.16–2.28	1.51*	1.16–1.96	1.97*	1.36–2.86
Low skill discretion	27	1.30	0.96–1.78	1.33	0.93–1.89	1.52*	1.15–2.02	1.93*	1.30–2.88
Poor relation with colleagues	28	1.40*	1.04–1.89	1.61*	1.14–2.26	1.16	0.89–1.53	1.70*	1.17–2.47
Poor relation with supervisor	28	1.71*	1.27–2.31	2.16*	1.53–3.05	1.28	0.98–1.68	1.78*	1.22–2.60

*Pe* prevalence in study population

^†^Reference category: no productivity loss

^‡^Reference category: no sick leave

* *p* < 0.05, adjusted for sex, age, and ethnicity

**Table 3:** Effects of adjustment for work-related factors, health, and lifestyle-related factors on the association between educational level and productivity loss at work (*n* = 647)

	10–20 % productivity loss^†^				30 % or more productivity loss^†^			
	Low education^‡^		Intermediate education^‡^		Low education^‡^		Intermediate education^‡^	
	OR	95 % CI	OR	95 % CI	OR	95 % CI	OR	95 % CI
Model 1: sex, age, and ethnicity	1.46*	1.01–2.11	1.22	0.89–1.67	1.49	0.98–2.26	1.28	0.87–1.87
Model 2: model 1 + reduced perceived general health	1.45*	1.00–2.08	1.21	0.88–1.65	1.43	0.94–2.19	1.28	0.87–1.87
Model 3: model 1 + work-related factors^a^	1.54*	1.06–2.23	1.24	0.90–1.70	1.54*	1.01–2.35	1.26	0.86–1.85
Model 4: model 1 + lifestyle-related factors^b^	1.46*	1.02–2.11	1.22	0.89–1.68	1.50	0.98–2.30	1.35	0.92–1.97
Model 5: model 1 + health + work-related factors	1.53*	1.05–2.21	1.23	0.90–1.70	1.49	0.97–2.28	1.27	0.86–1.86
Model 6: model 1 + health + work-related factors + lifestyle-related factors	1.53*	1.06–2.22	1.24	0.90–1.71	1.54*	1.01–2.37	1.32	0.90–1.94

^†^Reference category: no productivity loss

^‡^Reference category: high educational level

^a^Work-related factors: low job control, poor relation with colleagues, and poor relation with supervisor

^b^Lifestyle-related factors: insufficient vigorous physical activity

* *p* < 0.05

### Sick leave

As shown in Table 2, individuals with a low (OR = 1.81, 95 % CI 1.15–2.85) or intermediate educational level (OR = 1.85, 95 % CI 1.21–2.82) were more likely to have 10 or more workdays sick leave. Obesity was statistically significantly associated with more sick leave days after adjustment for gender, age, and ethnicity (OR = 2.29, 95 % CI 1.27–4.12). The strongest association was found between perceived general health and sick leave (OR = 6.26, 95 % CI 3.47–11.29). Several work-related factors were also associated with sick leave: working in awkward postures, low job control, low skill discretion, and a poor relation with colleagues or supervisor (Table 2). The combination of work-related factors partly explained the association between educational level and sick leave (Table 4). After adjustment for work-related factors, the strength of the association between a low educational level and 10 or more days of sick leave decreased from OR = 1.81 to OR = 1.62 (23 % change). Combined adjustment for work-related factors and perceived general health further reduced the strength of the association between a low educational level and 10 or more days of sick leave with an additional 4 %. After additional adjustment for overweight/obesity, the strength of the association between a low educational level and 10 or more days of sick leave further reduced with another 21 % (48 % change from OR = 1.81 to OR = 1.42).

**Table 4:** Effects of adjustment for work-related factors, health, and lifestyle-related factors on the association between educational level and sick leave

	1–9 days sick leave^†^				10 or more days sick leave^†^			
	Low education^‡^		Intermediate education^‡^		Low education^‡^		Intermediate education^‡^	
	OR	95 % CI	OR	95 % CI	OR	95 % CI	OR	95 % CI
Model 1: sex, age, and ethnicity	1.06	0.76–1.48	1.29	0.98–1.70	1.81*	1.15–2.85	1.85*	1.21–2.82
Model 2: model 1 + reduced perceived general health	1.07	0.77–1.50	1.30	0.99–1.72	1.77*	1.12–2.81	1.81*	1.18–2.79
Model 3: model 1 + work-related factors^a^	1.00	0.71–1.41	1.20	0.91–1.58	1.62*	1.01–2.61	1.69*	1.09–2.62
Model 4: model 1 + lifestyle-related factors^b^	1.04	0.74–1.47	1.29	0.97–1.71	1.69*	1.05–2.75	1.77*	1.14–2.77
Model 5: model 1 + work-related factors + health	1.04	0.74–1.47	1.22	0.92–1.62	1.59	0.99–2.55	1.65*	1.05–2.59
Model 6: model 1 + work-related factors + health + lifestyle-related factors	0.98	0.69–1.40	1.18	0.88–1.58	1.42	0.86–2.34	1.58	0.98–2.54

^†^Reference category: no sick leave

^‡^Reference category: high educational level

^a^Work-related factors: awkward postures, low job control, low skill discretion, poor relation with colleagues

^b^Lifestyle-related factors: overweight/obesity

* *p* < 0.05

## Discussion

In the current study, it was aimed to identify whether working conditions as well as lifestyle-related factors and health play a role in the causal pathway of educational inequalities in productivity loss at work and sick leave. Educational differences were found for productivity loss at work and sick leave. These educational differences in productivity loss at work and sick leave were particularly apparent in the more severe categories of productivity loss at work and sick leave. Unhealthy lifestyle-related factors, a poor general health, and unfavorable work conditions were also more prevalent among lower educated employees, but did not influence the association between education and productivity loss at work. Work-related factors and obesity did have an influence on educational differences in sick leave.

Previous research found educational differences in sick leave (Beemsterboer et al. 2009; Duijts et al. 2007). In our study, these findings were corroborated, especially for 10 or more days with sick leave. We also found educational differences in productivity loss at work. Employees with a low educational level had a higher risk of productivity loss at work. Although productivity loss at work and sick leave were not associated, educational level was associated with both outcomes.

The results of this study imply that both work-related and lifestyle-related factors do play a role in the mechanisms through which socioeconomic position affects sick leave. Unhealthy lifestyle behaviors and a decreased perceived general health were more prevalent among lower educated persons (see also Kamphuis et al. 2008; Kunst et al. 2005; Mackenbach et al. 2008). For productivity loss at work, these factors did not change the associations between educational levels and productivity loss at work. However, the association between sick leave and educational level decreased after adjustment for work-related and lifestyle-related factors. The relation between a poorer general health, on one hand, and productivity loss at work or sick leave, on the other hand, was consistent over the educational groups. Adjusting for health status between educational groups did not lead to a reduction in the strength of the association between educational level and productivity loss at work or sick leave. This implies that the higher prevalence of health problems among lower educated workers is not a major factor in the pathway between educational level and sick leave. In accordance with the study of Laaksonen et al. (2010a), work-related factors and overweight/obesity had the biggest influence on the relation between educational level and sick leave. However, in the study of Laaksonen et al. (2010a), strenuous physical work conditions instead of psychosocial work conditions provided the strongest explanation for socioeconomic differences in sickness absence. In contrast with other studies (Alavinia et al. 2009b; Laaksonen et al. 2010b; Lund et al. 2006), we did not find an association between having a physically demanding job and sick leave, nor between lifting heavy loads and sick leave. A possible explanation might be that the proportion of workers with exposure to mechanical load was low in our study population. Although 9 % was exposed to lifting heavy loads in our study, only 3 % answered ‘a lot’ on the question how often they have to lift heavy loads. This might indicate that those workers who were classified as having strenuous work conditions in our study are not that highly exposed to the specific physical work conditions. The evidence from literature indicates that both psychosocial and physical work-related factors may play a role in explaining educational differences in sick leave (Laaksonen et al. 2010a; Melchior et al. 2005; Niedhammer et al. 2008). Therefore, interventions aimed at improving work conditions, especially at postures, job control, and skill discretion, among lower educated employees might reduce educational differences in sick leave. However, a large proportion of the educational differences in sick leave could not be explained by these factors. Other factors, like coping strategy, social support, and motivation to work, were not measured in our study and may be relevant in explaining educational differences in sick leave, but also in productivity loss at work (Rael et al. 1995; Smith et al. 2008). In addition, factors like organizational problems, machine breakdown, or personal issues might particularly influence productivity loss at work. This might also be an explanation for the lack of influence of an unhealthy lifestyle and unfavorable working conditions on the association between education and productivity loss at work.

Relations between lifestyle-related factors and sick leave are well studied. In previous research, a relation between obesity and sick leave was found, especially with long-term sick leave (Alavinia et al. 2009b; Neovius et al. 2009; Robroek et al. 2011; Van Duijvenbode et al. 2009). Concerning productivity loss at work less evidence is available on the specific role of lifestyle-related factors. We observed an association between insufficient vigorous physical activity and more than 30 % productivity loss at work. However, this association was found only among better educated employees. A possible explanation might be found in the role of physical activity to reduce perceived stress. Vigorous physical activity may be a method to release stress in mentally demanding jobs and thereby decrease productivity loss at work (Hansen et al. 2010). It might be an interesting topic for future research to study whether physical activity buffers the relation between job demands and productivity loss at work in different types of work.

### Limitations

Firstly, participation levels differed between companies, partly because three companies had restricted the maximum participation. However, baseline participation levels (ranging from 36 to 61 %) in the other companies without restrictions were comparable with other studies on health promotion programs at the worksite, and in a systematic review, no evidence was found for selective participation concerning health or lifestyle indicators (Robroek et al. 2009). Secondly, subjective single measures of productivity loss at work and sick leave were used. There is ongoing discussion on how to measure productivity loss at work in a reliable and valid way (Koopmanschap et al. 2005; Zhang et al. 2011). Objective measures of productivity loss at work are rarely available, and the quantity question of the QQ method was associated with objective work output among floor layers (*r* = 0.48). A disadvantage of this method is that productivity loss is assessed during the previous regular workday and does not take into account the expected fluctuations in productivity loss within workers across workdays. Thirdly, as we described in the results, there is selective loss to follow-up. However, no selective loss to follow-up was found in the outcome measures. Fourthly, sickness absence has a multifactorial nature. Although we adjusted for several factors in the analyses, there may be confounders that were not taken into account. Last, self-reported health was measured with a single item. In a recent study, the reliability of the often used single question for general self-reported health was discussed. It was suggested that dichotomization may be a useful strategy for increasing the reliability of the measure in the total population (Zajacova and Dowd 2011).

## Conclusion

In conclusion, educational differences were observed in productivity loss at work and sick leave. These differences could hardly be assigned to health. Work-related and lifestyle-related factors did attenuate the association between low education and sick leave, but did not influence the association between educational level and productivity loss at work. These educational differences in sick leave prompt for interventions that address behavioral aspects as well as work-related and lifestyle-related factors.

## Abbreviations

PA: Physical activity
BMI: Body mass index
